# Snakebite and its impact in rural communities: The need for a One Health approach

**DOI:** 10.1371/journal.pntd.0007608

**Published:** 2019-09-26

**Authors:** Sara Babo Martins, Isabelle Bolon, François Chappuis, Nicolas Ray, Gabriel Alcoba, Carlos Ochoa, Sanjib Kumar Sharma, Armand S. Nkwescheu, Franck Wanda, Andrew M. Durso, Rafael Ruiz de Castañeda

**Affiliations:** 1 Institute of Global Health, Faculty of Medicine, University of Geneva, Geneva, Switzerland; 2 Division of Tropical and Humanitarian Medicine, Geneva University Hospitals, Geneva, Switzerland; 3 Institute for Environmental Sciences, GeoHealth group, University of Geneva, Geneva, Switzerland; 4 Médecins Sans Frontières, Geneva, Switzerland; 5 Department of Internal Medicine, B. P. Koirala Institute of Health Sciences, Dharan, Nepal; 6 Cameroon Society of Epidemiology, Yaoundé, Cameroon; 7 Centre d’Enseignement, Recherche, et Soins, Akonolinga, Cameroon; Faculty of Medicine, University of Kelaniya, SRI LANKA

Snakebite envenoming is a neglected tropical disease (NTD) with a significant public health impact. It is estimated to cause up to 138,000 deaths worldwide every year and 400,000 victims of permanent disability, including blindness or restricted mobility (reviewed in [1]). The most vulnerable populations are those where the presence of venomous snakes overlaps with the lack of access to healthcare and effective treatment [2].

In 2017, the World Health Organization (WHO) formally listed snakebite envenoming (snakebite hereafter) as a NTD, adding it to the global health agenda and marking an important shift in both awareness and control efforts [1]. In 2018, the resolution on snakebite envenoming adopted by the 71st World Health Assembly urged Member States to assess and address the burden of snakebite. In a topic area in which large data gaps remain, there is currently also a call for a wider, transdisciplinary approach to the snakebite problem and overall enhanced systemic thinking in the field [3].

This viewpoint highlights the need to frame snakebite as an issue at the interface of humans, domestic animals, and snakes in agroecosystems, in such a systemic thinking context. We explore how human health, animal health, and reliance on agricultural activity and domestic animals for livelihood should be considered in a One Health approach. We further discuss how One Health can be used to enhance our understanding of this complex issue, namely of its eco-epidemiology and its broad socioeconomic impact, contributing to filling key research gaps.

## Snakebite and the interface of humans, domestic animals, and snakes in agroecosystems

Snakebite is often described as an occupational disease, particularly affecting farmers, cattle herders, and other agricultural workers in poor rural communities [1]. Globally, mortality has been shown to be strongly correlated with the percentage of workforce in agriculture [4].

Certain characteristics of agroecosystems are key determinants of the human–livestock–snake conflict, and some agricultural practices can intensify snakebite risk. For example, the increasing number of large-scale, industrial plantations of palm, sugar cane, banana, or rubber in the tropics may offer new ecological niches for some disturbance-tolerant species of snakes to exploit and proliferate [5,6]. In India, spectacled cobras (*Naja naja*) and monocellate cobras (*N*. *kaouthia*) are commonly associated with paddy fields [7]. In Brazil, a positive correlation exists between snakebite incidence and cocoa and coffee plantations, as well as bovine and domestic chicken breeding [8].

In these agricultural contexts, snakebite also causes mortality and morbidity of domestic animals. In central Costa Rica, terciopelos (*Bothrops asper*) are estimated to cause approximately 10,000 cattle envenoming cases per year, prompting the development of a toxoid vaccine [9]. This problem extends to other regions of the world, as identified in a recent scoping review [10]. Various types of domestic animals, including pets, horses, goats, cattle, and camelids, have been reported to be affected by venomous snakes, causing fatality rates of above 47% in livestock. Most of the snake species identified in the aforementioned review are medically important venomous snakes (according to WHO) that also threaten human lives and their livelihoods [10].

Domestic animals are an important part of the livelihoods of communities in rural settings. About half of the world’s poor population fully or partly depend on livestock for their livelihoods [11]. Keeping livestock provides subsistence consumption of meat or dairy, supports other activities (such as crop production), protects against seasonality in income, provides assets for insurance, and may also enable advancement to other livelihood activities [12]. Losses of domestic animals can therefore represent an important socioeconomic impact in affected communities. In addition, domestic animal losses can have an important emotional impact.

## A One Health approach to snakebite

The coexistence between humans, their domestic animals, and snakes and the subsequent impact of snakebite in rural communities are complex and depend on multiple ecological (e.g., snake diversity, distribution), cultural, and socioeconomic factors (e.g., type of agricultural practices, type of domestic animals). However, to date, little attention has been given to snakebite with a systemic socioecological perspective that also includes domestic animals.

A One Health approach, which recognizes the interlinkages among humans and their domestic animals with snakes in a wider context, can be valuable in a number of ways. It provides further understanding of the eco-epidemiology of snakebite in humans, particularly the role of domestic animals as a risk or protective factor. It also addresses data gaps surrounding the impact of snakebite in animal health. In other NTDs, notably in dog-mediated rabies, a One Health approach has been used to generate robust evidence about the epidemiology of transmission and disease burden and, importantly, has underpinned the design and implementation of successful and cost-effective control programs through dog vaccination [13].

A One Health approach can further contribute to a more accurate assessment of the socioeconomic impact of snakebite in affected communities. In the human health dimension, available national estimates place snakebite as a leading cause of health burden due to NTDs in terms of disability-adjusted life years [14]. The literature on socioeconomic aspects of snakebite has also explored the expenditure associated with snakebite treatment [15,16] and has estimated the cost-effectiveness of antivenom treatment (reviewed in [17]). This body of evidence clearly identifies the importance of snakebite as a human health threat and the financial toll associated with its clinical management. The impact of snakebite in affected communities could be even higher if livelihood losses associated with domestic animal cases are considered.

**Fig 1:**
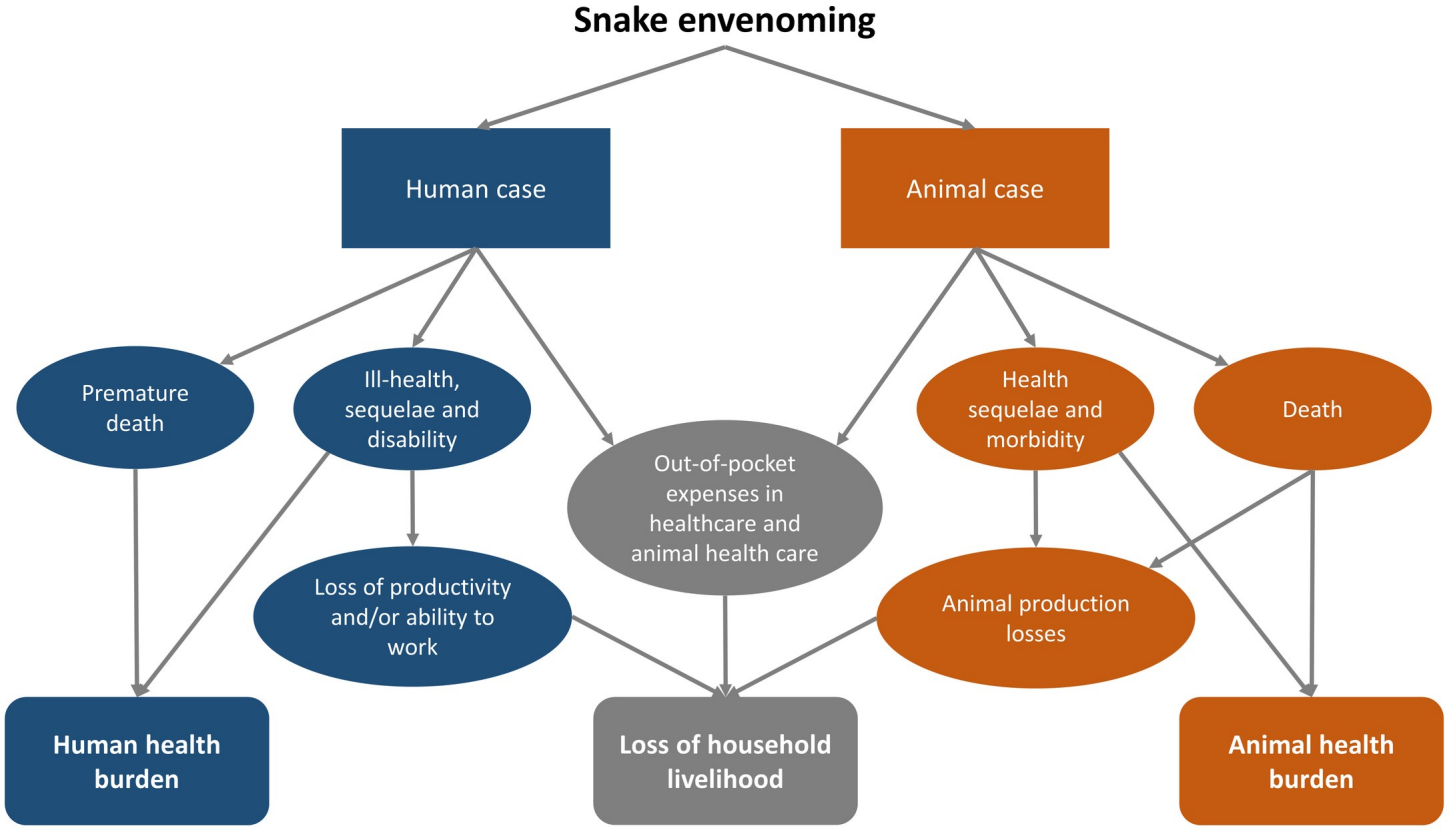
Pathways to health and livelihood losses due to snakebite in a One Health approach to the socioeconomic impact assessment of snakebite.

This double impact linked to human health and livelihoods can be captured through the pathways for losses conceptualized in Fig 1. The overall socioeconomic impact of snakebite considers losses associated with premature death and ill-health sequelae, loss of productivity and ability to work, and healthcare expenses associated with human cases. To this burden are added livelihood losses associated with the death of animals, health sequelae and morbidity that can impair productivity, and expenditure associated with animal health in affected households.

A One Health approach to snakebite and its impact is currently being implemented in the ongoing 4-year SNAKE-BYTE project (2018–2022). The project has been designed to improve our understanding of snakebite burden, its impact on livelihoods, and the physical accessibility of existing human and animal health services, through national cross-sectoral data collection at the household level in two representative snakebite endemic countries—Cameroon and Nepal. In this project, primary data on human and domestic animal mortality and morbidity, symptoms and outcomes, healthcare costs, and livestock production losses are collected jointly in a survey covering 12,000 households in each country.

Building on a solid foundation of primary data and transdisciplinarity, this work will add new evidence on the assessment of risk factors linked with animal ownership for human cases and on the epidemiology of animal cases. It will also generate an assessment of the socioeconomic impact of snakebite in affected communities in the target countries, considering the pathways for health and livelihood losses summarized previously. This information can be key in raising awareness on the impact of snakebite, in shaping policy making, and in implementing effective field interventions, including access to vital antivenoms, particularly relevant in the case of snakebite.

A One Health approach to snakebite encompasses the recognition of the interface of humans, domestic animals, and snakes in agroecosystems. It also includes the recognition of how this interface can be significant in the eco-epidemiology and impact of snakebite in human and animal health. Such systemic thinking and transdisciplinarity, including the involvement of stakeholders from a range of fields such as agriculture, ecology, animal health, herpetology, forestry, anthropology, and education, are key in achieving the goals recently set out by the global health community in the fight against snakebite [18].
